# Improving Women's Sexual and Reproductive Health in Acute Inpatient Psychiatric Services – A Quality Improvement Project

**DOI:** 10.1192/bjo.2024.446

**Published:** 2024-08-01

**Authors:** Sally Tulip, Sarah Godsell, Sophie Roche, Alara Acunas Okay, Kirsty Hartshorn

**Affiliations:** 1 Barnet, Enfield and Haringey Mental Health Trust, London, United Kingdom; 2 North Middlesex University Hospital NHS Trust, London, United Kingdom

## Abstract

**Aims:**

Women with severe mental illness are at higher risk of sexually transmitted infections (STIs), unplanned pregnancies and poor engagement with cervical and breast screening. Despite current national guidance, these issues are poorly addressed during psychiatric admissions.

We aimed to improve the provision of women's sexual and reproductive healthcare on psychiatric wards using a quality improvement framework.

**Methods:**

Female psychiatric inpatients aged over 18 were included. A baseline audit was performed in October 2022 on a female psychiatric ward, followed by six PDSA cycles from August 2022–January 2024 (n = 108).

We introduced women's health assessments (WHAs), offering counselling on: (1) contraception, (2) cervical and breast screening, and (3) STI screening. We arranged treatment and follow-up.

Changes were made at each PDSA cycle: ensuring provision of emergency contraception and STI swabs; establishing a protocol for referring to the sexual health clinic; creating dedicated clinic time to offer counselling; developing a poster and educational leaflet; and creating a proforma to record outcomes. The interventions were then extended to a neighbouring ward.

We reviewed electronic notes and recorded the percentage of patients offered counselling at baseline and after each cycle, later also recording the percentage of patients accepting interventions.

**Results:**

At baseline, 12.5% of inpatients had been offered at least one of: contraceptive counselling, cervical and breast screening or STI screening. This improved to 87.7% offered a leaflet and 63.1% offered counselling by the final cycle. Of these patients, 48.8% accepted at least one intervention. On the neighbouring ward, offers of counselling increased from 28.6% to 63.6%.

Introduction of dedicated clinic time increased offers of interventions the most, to 94.1% (cycle 3). Compliance was lowest in cycle 4 (54.2% offered any intervention) which coincided with junior doctor changeover. Provision of an educational leaflet did not increase acceptance of interventions (cycle 5).

Introduction of WHAs led to detection and treatment of STIs in seven patients. Absent contraception was identified and started for a patient taking sodium valproate. Five patients were administered emergency contraception and two commenced long-term contraceptives. A case of female genital mutilation was identified, and a case of cervical neoplasia (CIN 3) was detected.

**Conclusion:**

Provision of WHAs improved women's healthcare in inpatient psychiatric settings, with clinician contact being the most valuable resource in achieving this. There were several barriers, importantly clinician availability and awareness during junior doctor changeover. We will establish our interventions trust-wide, protocolising WHAs in the junior doctors’ handbook, and collect patient feedback.

Additional authors: Dr Terteel Elawad, Dr Judith Stellman.

